# Developing a composite outcome measure for frailty prevention trials – rationale, derivation and sample size comparison with other candidate measures

**DOI:** 10.1186/s12877-020-1463-x

**Published:** 2020-03-25

**Authors:** Miles D. Witham, James Wason, Richard Dodds, Avan A. Sayer

**Affiliations:** 1 AGE Research Group, NIHR Newcastle Biomedical Research Centre, Translational and Clinical Research Institute, Faculty of Medical Sciences, Newcastle University and Newcastle Upon Tyne Hospitals NHS Foundation Trust, Newcastle-upon-Tyne, NE4 5PL UK; 2 Population Health Sciences Institute, Faculty of Medical Sciences, Newcastle University, Newcastle-upon-Tyne, UK

**Keywords:** Trials, Frailty, Outcomes, Sample size, Markov

## Abstract

**Background:**

Frailty is the loss of ability to withstand a physiological stressor and is associated with multiple adverse outcomes in older people. Trials to prevent or ameliorate frailty are in their infancy. A range of different outcome measures have been proposed, but current measures require either large sample sizes, long follow-up, or do not directly measure the construct of frailty.

**Methods:**

We propose a composite outcome for frailty prevention trials, comprising progression to the frail state, death, or being too unwell to continue in a trial. To determine likely event rates, we used data from the English Longitudinal Study for Ageing, collected 4 years apart. We calculated transition rates between non-frail, prefrail, frail or loss to follow up due to death or illness. We used Markov state transition models to interpolate one- and two-year transition rates and performed sample size calculations for a range of differences in transition rates using simple and composite outcomes.

**Results:**

The frailty category was calculable for 4650 individuals at baseline (2226 non-frail, 1907 prefrail, 517 frail); at follow up, 1282 were non-frail, 1108 were prefrail, 318 were frail and 1936 had dropped out or were unable to complete all tests for frailty. Transition probabilities for those prefrail at baseline, measured at wave 4 were respectively 0.176, 0.286, 0.096 and 0.442 to non-frail, prefrail, frail and dead/dropped out. Interpolated transition probabilities were 0.159, 0.494, 0.113 and 0.234 at two years, and 0.108, 0.688, 0.087 and 0.117 at one year. Required sample sizes for a two-year outcome in a two-arm trial were between 1040 and 7242 for transition from prefrailty to frailty alone, 246 to 1630 for transition to the composite measure, and 76 to 354 using the composite measure with an ordinal logistic regression approach.

**Conclusion:**

Use of a composite outcome for frailty trials offers reduced sample sizes and could ameliorate the effect of high loss to follow up inherent in such trials due to death and illness.

## Background

Frailty is a state of decreased reserve such that a minor perturbation (such as a mild illness or injury) causes major physiological decompensation and worsening of health [1]. Frailty predicts multiple adverse outcomes in older people. Including falls, hospitalisation, disability, a need for care, and earlier death. Although long recognised as a clinical concept, frailty was not effectively operationalised until the early 2000s [2, 3]. Since then, uptake of frailty measures into clinical practice has increased rapidly, both in specialist services for older people, but more recently in primary care services and in organ-specific specialist services [4, 5]. Despite this widespread assessment of frailty in clinical practice, much less is known about what interventions are effective in reversing, or preventing progression of, the frailty state. Comparatively few randomised controlled trials have been conducted in this area to date, and most trials have focussed on exercise-based programmes [6–9].

Perhaps understandably given the nascent nature of this research field, there is no consensus on the most appropriate outcome measure to use in frailty intervention trials – particularly in early-phase trials that are essential in identifying the most promising candidate interventions to take forward to large-scale testing. Measures of physical performance have been used in some trials; examples include the Short Physical Performance Battery (SPPB), which has been advocated by the SPRINTT consortium as a surrogate for identifying frailty [10, 11] and gait speed, used in a recent trial of metformin in prefrail individuals [12] and as a secondary outcome in a recent exercise trial [13]. An alternative approach is to use measures of activity limitation as the main outcome. Inability to complete a 400 m walk within 15 min was the primary outcome for the SPRINTT trial, with the authors arguing that this measure denotes progression of frailty to disability. Such approaches have the advantage of using well-characterised, simple measures, but they depart conceptually from the original definition of frailty – “a multidimensional syndrome characterised by decreased reserve and diminished resistance to stressors” [14]. Use of single physical performance measures such as the SPPB to define frailty restricts the definition of frailty to impairment of neuromuscular function. Even use of an aerobic measure of physical performance (such as the 6 min walk or 400 m walk), which has the advantage of including cardiorespiratory function, does not encompass other aspects of the frailty syndrome such as impaired energy homeostasis or multisystem dysfunction.

There are two main ways of operationalising the frailty syndrome that might be used as direct outcome measures in clinical trials. The first is the deficit accumulation index [3]. This can now be assessed by automated means from routinely collected clinical data [15] but many of the deficits from which the index is built are based on diagnoses – and are thus unlikely to be reversed by intervention. Deficit accumulation indices may therefore be a useful way of *identifying* those with the frailty syndrome but may not be useful as an *outcome* measure – particularly over short time periods. The alternative is to use a measure of phenotypic frailty, of which the Fried frailty score is the most widely used [2]. Whilst physical performance measures form part of this score, they are complemented by a measure of energy homeostasis (weight loss), activity, and exhaustion – all of which more closely reflect the impairment of whole-body homeostasis alluded to in consensus definitions of frailty.

Two important issues complicate the use of frailty as measured by the Fried phenotype. Firstly, transition rates from prefrailty to frailty are relatively low (18% over 4 years in a recent meta-analysis) [16], thus large sample sizes are likely to be required to detect differences in transition rates. Furthermore, transition rates from frailty to death are high – thus the time spent in the frail state may be comparatively short, and dropout rates (due to death or illness) are thus also likely to be high. Treating the Fried phenotypic score as a continuous variable has been used as an alternative approach, although it is questionable whether this is appropriate way to analyse such a variable given that a one-unit step may not be a constant quantity, and may vary depending on what components are included and where in the score distribution the value is located. Even using this approach, sample sizes of 500–600 are required to detect a one-point difference [17].

To enable sample size calculations to be conducted for frailty prevention trials, data are required on rates of transition between prefrailty and frailty in target populations under a range of assumptions. The aim of this paper is to calculate these transition rates using data from the English Longitudinal Study of Ageing (ELSA), and to compare required sample sizes for simple frailty transitions, a composite measure of progression to frailty, death or dropout.

## Methods

### Derivation of transition rates for different frailty states

To study transition rates between robust, prefrail and frail states, we calculated phenotypic frailty scores after the method of Fried et al. [2] using data from ELSA waves 2 (collected 2004) and 4 (collected 2008) [18]. We included participants aged 60 and over at the time of the wave 2 data collection. Participants in these waves underwent a nurse-led assessment, at which grip strength, weight and gait speed were collected allowing a phenotypic frailty score to be derived. The phenotypic frailty score was derived using the same methods as previously used in ELSA, using the components first described by Fried et al. [2, 19]. Detailed methods for deriving each component of the frailty score are given in Table 1. Maximal handgrip strength was measured using a Smedley dynamometer, with results adjusted for body mass index. Walk speed was measured over a four metre course. Two attempts were recorded, with the highest walk speed used and adjusted for height. Weight was measured to the nearest kilogram, and the difference in weight between waves 0 and 2 was calculated. A physical activity index was calculated from self-reported frequency of mild, moderate and vigorous activity, with weights of 1.5, 3 and 6 METS respectively [20], multiplied by frequency for each activity category: Never (× 0), 1–3 times per month (× 2), once a week (× 4) or more than once a week (× 8). This approach allowed a more finely graduated estimate of physical activity than the derived four-state summary variable available in the ELSA dataset. Finally, self-reported exhaustion was measured using answers to two questions from the CES-D questionnaire: ‘everything I did was an effort’ and ‘I could not get going’. One point was awarded to those in the lowest sex-specific fifth for adjusted grip strength, adjusted walk speed and self-reported activity; one point was awarded if either self-reported exhaustion question was positive, and one point was awarded if the BMI was < 18.5 kg/m^2^ or for > 10% weight loss since the last measurement in ELSA. This dual approach for weight was adopted to minimise the impact of missing prior weight measurements. In all cases, the thresholds for defining each component were derived from the wave 2 data and applied without change to the wave 4 data. Measurements on all five frailty components had to be available at Wave 2 for an individual to have a frailty score calculated and to be included in the analyses. This approach is necessary for accurate coding of prefrailty, and also most accurately reflects the data state likely to be present at enrolment into frailty trials.

**Table 1:** Derivation of frailty score components in ELSA

Component	Definition
Weight loss	BMI < 18.5 kg/m^2^ OR > 10% weight loss since last wave
Exhaustion	Positive answer to either CES-D question (‘everything I did was an effort’ or ‘I could not get going’
Low grip strength (adjusted for BMI) Males: Grip adjusted for BMI = Maximum grip – ((BMI-27.6)× 0.31) Females: Grip adjusted for BMI = Maximum grip – ((BMI-27.9)× 0.07)	Low grip strength was defined as values in the lowest quintile at wave 2, for males and females separately: M < 31.12 kg F < 17.60 kg
Low gait speed (adjusted for height) Males: Gait speed adjusted for BMI = Gait speed – ((height-172)×0.00941) Females: Gait speed adjusted for BMI = Gait speed – ((height-158)× 0.010)	Low gait speed was defined as values in the lowest quintile at wave 2, for males and females separately. M < 0.691 m/s F < 0.619 m/s
Low activity	M < 16.5 activity units F < 13.5 activity units

### Analyses - frailty transitions in ELSA

For all analyses, only those individuals with complete data for all five components of the frailty score were included. This choice is likely to exclude some people who are particularly frail or unwell (as shown in other studies of phenotypic frailty scores) [21], but it reflects more accurately the population likely to enter trials to treat frailty or prefrailty (for whom a complete frailty score at baseline would be obtained). Summary statistics for this dataset were calculated using SPSS v22 (IBM, New York, USA).

### Analyses - interpolation of transition rates at 1 and 2 years

**Fig. 1:**
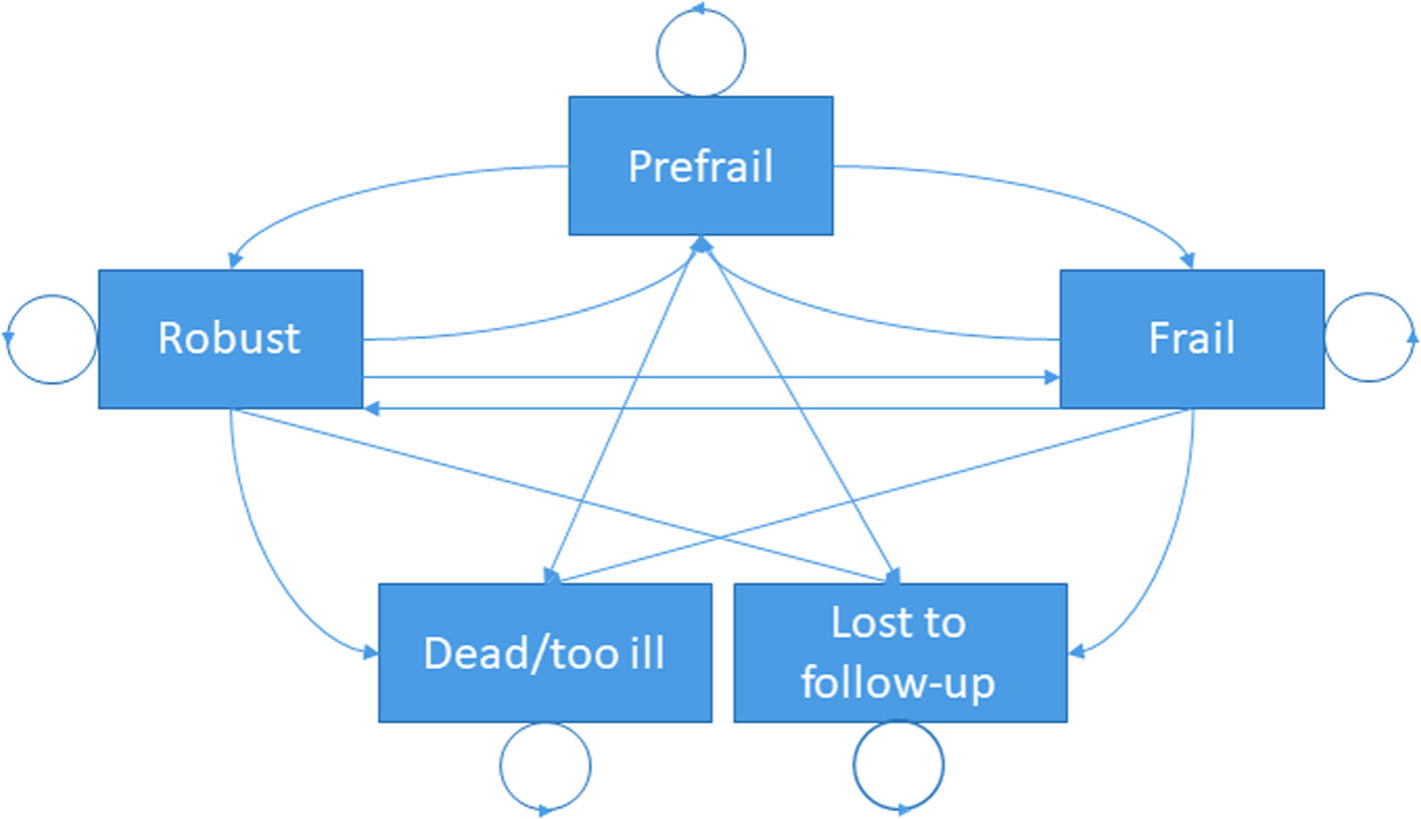
Markov model for frailty state transitions

The span between waves where the frailty phenotype was evaluable in ELSA was 4 years. Given the expense of conducting four-year follow up in trials, allied to the high dropout rate over this time period, shorter follow up times (1 or 2 years) are desirable for frailty trials. To interpolate the transition rates at 1 and 2 years, a Markov state transition model was constructed (Fig. 1) with probabilities fitted from the ELSA 4 year follow up data. The square root of the transition probability matrix was calculated to derive two-year transition probabilities, and the fourth-root of the transition probability matrix was calculated to derive the one-year transition probabilities.

We defined P as the transition matrix for the four-year transition probabilities with P_ij, i, j∈{1,2,3,4,5} representing the probability of transitioning from state i to state j in 4 years. The states were robust (1), prefrail (2), frail (3), dead or too ill to continue in study (4), dropped out for reasons other than being too ill (5). We then used the matrix decomposition to express P as ZAZ^(− 1) where Z is a matrix with the eigenvectors of P as the columns, and A is a diagonal matrix with (positive) eigenvalues along the diagonal. This allowed us to find matrix square- and fourth-root as ZA^(1/2) Z^(− 1) and ZA^(1/4) Z^(− 1) respectively [22]. These were taken as estimates of the one- and two-year transition probabilities and the second row of each extracted as the transition probabilities for pre-frail patients. This process was performed in R version 3.5 using the functions ‘eigen’ to extract the eigenvectors and eigenvalues of the original four-year transition matrix, and ‘ginv’ to find the inverse of the matrix of eigenvectors. It was not possible from ELSA data to determine whether participants who dropped out did so because they were too ill or for other reasons. We therefore considered three potential assumptions for participants who dropped out: 1) all dropout was due to being too ill to continue; 2) 50% of dropout was due to being too ill; 3) all dropout was for reasons other than being too ill.

### Analyses - calculation of sample sizes

For all sample size calculations, a two-sided alpha of 0.05 and a power of 0.90 was used. No consensus exists for the degree of reduction in the risk of transition to frailty that is clinically important, thus values for a range of relative risk reductions were calculated. Sample sizes for ordinal regression approach were calculated using the method of Whitehead [23]. To provide further comparisons with sample sizes for simple and composite frailty outcomes, we derived sample size calculations for the Fried frailty score used as a continuous measure, the four metre walk speed and the SPPB, which are continuous variables that have been proposed for use as outcome measures in frailty trials and are recommended as measures of physical performance for use in clinical practice [24]. For the Fried score, no anchor-based MCID has been proposed. We therefore assume a between-group difference of 0.3 points as seen in Serra-Prat et al. [13], and a SD of 1.2 points at follow up for those who were prefrail at wave 2 in ELSA. The MCID for the four-metre walk distance has been estimated, with values between 0.05 m/s and 0.10 m/s [25]. We used both these values, with a SD of 0.25 m/s (based on values from ELSA at wave 4 for those who were prefrail at wave 2, and consistent with other trials for prefrailty [14, 15]. Finally, the MCID for the SPPB has been estimated at 1 point [26] or 0.5 points [25]. We used both these values, together with an SD of 2.5 as seen in a previous trial conducted with functionally impaired older people [27]. Sample sizes were calculated with and without adjustment for baseline values; a correlation of 0.5 between baseline and follow up was assumed for adjusted analyses which is conservative (i.e. lower) than that observed in previous trials using walk speed or SPPB.

## Results

### Transition probabilities from ELSA data

Data on 5344 participants from wave 2 aged 60 and over were available; not all participants had data available for each frailty component, and a complete frailty score was calculable for 4650 individuals in wave 2, who thus formed the analysis sample. 390/4650 (8.4%) died between wave 2 and wave 4; 3088/4650 (66.4%) underwent and completed the wave 4 assessment. Details of frailty components and Fried frailty scores at wave 2 and wave 4 are given in Tables 2 and 3 along with age and sex of the study population and details of missing data for each frailty score component.

**Table 2:** Prevalence of frailty components in wave 2 and wave 4 of ELSA

		Wave 2 (*n* = 5344)	Wave 4 (*n* = 3088)
Mean age (years) (SD)	All	71.3 (8.2)	69.9 (7.2)
	With any missing data	74.2 (9.7)	73.9 (8.3)
Male sex (%)	All	2389 (44.7)	1368 (44.3)
	With any missing data	299/694 (43.0)	138/374 (36.9)
Weight loss / low BMI (%)	Calculable	391/5107 (7.7)	282/3088 (9.1)
	Missing	237/5344 (4.4)	0/3088 (0)
Low grip strength (%)	Calculable	1049/5251 (20.0)	689/2888 (23.9)
	Missing	93/5344 (1.7)	200/3088 (6.5)
Low gait speed (%)	Calculable	977/4886 (20.0)	694/2880 (24.1)
	Missing	458/5344 (8.6)	208/3088 (6.7)
Exhaustion (%)	Calculable	1638/5314 (30.8)	816/3077 (26.5)
	Missing	30/5344 (0.6)	11/3088 (0.4)
Low activity levels (%)	Calculable	1196/5341 (22.4)	709/3088 (23.0)
	Missing	3/5344 (0.06)	0/3088 (0)

**Table 3:** Prevalence of Fried frailty scores in wave 2 and wave 4 of ELSA

Fried Frailty score		Wave 2 (*N* = 4650)		Wave 4 (*N* = 2714)	
Mean age (years) (SD)		70.8 (7.9)		69.3 (6.9)	
Male sex (%)		2560 (55.1)		1230 (45.3)	
0 (%)	*Robust (%)*		*2226 (47.9)*		*1282 (47.2)*
1 (%)	*Prefrail (%)*	1287 (27.7)	*1907 (41.0)*	747 (27.5)	*1108 (40.8)*
2 (%)		620 (13.3)		361 (13.3)	
3 (%)	*Frail (%)*	349 (7.5)	*517 (11.1)*	223 (8.2)	*324 (11.9)*
4 (%)		150 (3.2)		85 (3.1)	
5 (%)		18 (0.4)		16 (0.6)	

Tables 4 and 5 give the transition probabilities between wave 2 and wave 4 of ELSA for different baseline frailty scores and different baseline frailty categories. Of particular note are the high dropout and death rates in those with frailty at baseline, and the low rates of reversion from frailty to the robust state at the four-year follow-up.

**Table 4:** Change in frailty state between wave 2 and wave 4 of ELSA

		Follow up frailty state at Wave 4				
Baseline state at Wave 2	n	Robust	Prefrail	Frail	Dead	Dropped out
Robust	2226	937 (42)	519 (23)	51 (2)	79 (4)	640 (29)
Prefrail	1907	335 (18)	546 (29)	189 (10)	178 (9)	659 (35)
Frail	517	10 (2)	43 (8)	84 (16)	133 (26)	247 (48)

**Table 5:** Changes in frailty score between wave 2 and wave 4 of ELSA

		Follow up frailty score at Wave 4							
Baseline score at Wave 2	n	0	1	2	3	4	5	Dead	Dropped out
0	2226	937 (42)	407 (18)	112 (5)	37 (2)	13 (1)	1 (0)	79 (4)	640 (29)
1	1287	289 (23)	259 (20)	139 (11)	83 (6)	15 (1)	2 (0)	95 (7)	405 (31)
2	620	46 (7)	67 (11)	81 (13)	65 (11)	17 (3)	7 (1)	83 (13)	254 (41)
3	349	8 (2)	13 (4)	24 (7)	30 (9)	22 (6)	2 (1)	82 (23)	168 (48)
4	150	2 (1)	1 (1)	5 (3)	8 (5)	17 (11)	3 (2)	45 (30)	69 (46)
5	18	0 (0)	0 (0)	0 (0)	0 (0)	1 (6)	1 (6)	6 (33)	10 (56)

### Interpolated transition probabilities

Table 6 gives the derived Markov transition probabilities from baseline prefrailty at 1 and 2 years under three assumed scenarios – firstly that all dropouts are due to illness, secondly that no dropouts are due to illness, and thirdly that half the dropouts were due to illness. Rates of transition to frailty are low at 1 year (9%) and at 2 years (11%) under all scenarios; rates of combined transition to frailty, death or dropout due to illness are still relatively low at 1 year (11 to 21%) but reach 16 to 35% at 2 years depending on the assumptions made about causes of dropout.

**Table 6:** Interpolated frailty state transition probabilities at one and two years from baseline prefrail state

Baseline state	Follow up state	1 year (interpolated)	2 year (interpolated)	4 year (measured)
Assumes all dropouts are due to illness	Robust	0.108	0.159	0.176
	Prefrail	0.688	0.494	0.286
	Frail	0.087	0.113	0.099
	Dead/too ill	0.117	0.234	0.439
Assumes no dropouts are due to illness	Robust	0.109	0.159	0.176
	Prefrail	0.688	0.494	0.286
	Frail	0.087	0.113	0.099
	Dead	0.019	0.044	0.093
	Lost to follow up	0.097	0.190	0.346
Assumes 50% of dropouts are due to illness	Robust	0.109	0.159	0.176
	Prefrail	0.688	0.494	0.286
	Frail	0.088	0.113	0.099
	Dead/too ill	0.068	0.139	0.266
	Lost to follow up	0.049	0.095	0.173

### Illustrative sample size calculations

Table 7 shows illustrative sample size calculations for a hypothetical trial enrolling people with prefrailty at baseline with a two-year follow up period. Results for different approaches to frailty transition are presented with a range of relative risk reductions as the minimum clinically important difference in frailty transition rate has not been established. For comparison, results of sample size calculations for the Fried frailty score as a continuous variable, the four-metre walk speed, and the SPPB are shown in Table 8. Projected sample sizes needed using a composite outcome of transition to death, frailty or dropout due to illness are less than a quarter of those needed to detect a simple transition to the frail state, and sample sizes needed are reduced by a further factor of three when using an ordinal regression approach to analysis that uses all transitions including from prefrail to non-frail status.

**Table 7:** Illustrative sample size calculations; transition from baseline prefrail status; 2 year follow up

To detect relative risk reduction of:	Transition to frail (per group)	Transition to frail/dead/too ill (per group)	Ordinal (robust vs prefrail vs non frail/dead/too ill (per group)
20%	3621	815	177
25%	2389	558	117
30%	1576	360	87
40%	853	197	55
50%	520	123	38

All two-sided, alpha 0.05, power 0.90

**Table 8:** Illustrative sample size calculations for continuous measures

Measure	MCID	SD	Unadjusted (per group)	Adjusted (per group)
Fried frailty score	0.3^a^	1.2	352	264
4 m walk speed	0.05	0.25	526	395
4 m walk speed	0.10	0.25	132	99
SPPB	0.5	2.5	526	395
SPPB	1.0	2.5	132	99

All two-sided, alpha 0.05, power 0.90, with correlation of 0.5 between baseline and follow up measures

^a^MCID unknown, but value of 0.3 derived from change seen in trial by Serra-Prat et al. [10]

*SPPB* Short Physical Performance Battery

## Discussion

### Findings in context

Our results from a large, representative sample of older people confirm that transition rates to the frail state from the robust or prefrail states are low, and that those in the frail state do not remain under follow-up, instead transitioning to death, or dropping out of follow up because of frailty and illness. Sample sizes required for trials using frailty transitions as an outcome are therefore very large, but the use of a composite outcome of transition to frailty, death or dropout allows a considerable reduction in the sample size required, whilst also ensuring that those who die or drop out still contribute to the analysis. Sample sizes using the composite outcome in an ordinal logistic regression analysis were comparable to, or lower than those needed for commonly-used measures of physical performance, and were lower than those required when using the Fried frailty score as a continuous variable.

The transition rates that we observed were compatible with previous findings; a recent meta-analysis with a mean 3.9 year follow up found higher rates of transition from prefrail to frail (18% compared to our finding of 10%) [16], but transition rates were heavily dependent on the length of follow-up. Our one-year interpolated estimates for transition from prefrailty to frailty were similar to that observed at 1 year follow up in the control arm of a trial enrolling participants with prefrailty [13], suggesting that our results would be applicable to trials for this patient group.

Conducting trials for older people poses specific challenges over and above those usually encountered by trialists. Recruitment can be challenging due to ageism, illness and logistical barriers [28], dropout rates are high, and multimorbidity and heterogeneity increase variance – hence sample sizes may need to be increased. Recruitment from institutional care settings poses additional challenges, including high death rates, high levels of cognitive impairment that complicate the process of consent, and organisational challenges in delivering research outwith traditional health service structures [29]. Finding outcome measures that can detect the effect of an intervention without a long period of follow-up or large numbers of participants is therefore challenging, but essential if the volume of trials conducted for older people is to meet the clinical needs of older people [30]. The proposed composite endpoint developed here addresses both the issue of sample size and the issue of loss to follow up due to death or illness, but also has the advantage of directly measuring the multidimensional construct of frailty. Measuring frailty transition rates provides an outcome measure that reflects the longitudinal progression of frailty experienced by patients. Modifying the natural history of frailty progression is a key objective of research and practice in this area, as highlighted by the European Union AdvantAGE joint action plan on frailty (https://advantageja.eu) and a frailty transition outcome measure could naturally facilitate conversations between clinicians and patients about whether to engage with different interventions to prevent or ameliorate frailty.

Some limitations require comment. We used a large, representative UK-based cohort to derive transition rates, and these rates are likely to vary across different countries and different cohorts. In particular, differences in age, sex and burden of comorbid disease are likely to be associated with variation in prevalence of frailty and different rates of frailty transition. However, these differences do not invalidate the concept of using a composite measure. We were unable to differentiate between those dropping out of ELSA due to illness and those dropping out from choice; our analyses assume that the majority dropped out due to illness or disability. The higher rates of dropout seen with worse baseline frailty status support this assumption, as do the higher dropout rates seen with increasing age and the presence of a limiting illness previously noted in the ELSA cohort [19]. Similarly, we do not have sufficient information from ELSA to tell whether dropout was due to physical illness or frailty, or due to progressive cognitive impairment. The four-year gap between waves 2 and 4 of ELSA may conceal multiple transitions in frailty states, in keeping with the dynamic nature of the frailty construct. This lack of temporal resolution can be only partly overcome by the Markov interpolation method that we used, and our proposed outcome needs to be tested prospectively in a frailty trial to fully reflect these dynamic changes.

## Conclusions

Use of a composite outcome for frailty trials offers reduced sample sizes and could ameliorate the effect of high loss to follow up inherent in such trials due to death and illness. As a next step, the acceptability of the composite endpoint to both people living with frailty or prefrailty, and to clinicians caring for this group of patients should be established. The outcome will be of use in shared decision making only if it carries meaning to those who will use the evidence. Secondly, the degree of change in transition rates that would change the decision to recommend a treatment should be established. This metric – similar to the MCID for continuous outcomes, is poorly defined for most categorical measures used in trials yet is essential to derive meaningful sample size calculations. Finally, the composite outcome should be applied to existing datasets from frailty trials to test how the proposed composite measure might work in the real world. Successful practical application and user acceptability will confirm or refute the usefulness of this approach.

## Data Availability

Data from the English Longitudinal Study of Ageing is freely available from https://www.elsa-project.ac.uk/

## Abbreviations

BMI: Body Mass Index
CES-D: Centre for Epidemiological Studies – Depression scale
ELSA: English Longitudinal Study of Ageing
MCID: Minimum Clinically Important Difference
SPPB: Short Physical Performance Battery
